# Age and severity-dependent gut microbiota alterations in Tunisian children with autism spectrum disorder

**DOI:** 10.1038/s41598-023-45534-0

**Published:** 2023-10-25

**Authors:** Mariem Chamtouri, Naoufel Gaddour, Abderrahmen Merghni, Maha Mastouri, Silvia Arboleya, Clara G. de los Reyes-Gavilán

**Affiliations:** 1 Department of Microbiology and Biochemistry of Dairy Products, Instituto de Productos Lácteos de Asturias (IPLA-CSIC), 33300 Villaviciosa, Spain; 2 Laboratory of Transmissible Diseases and Biologically Active Substances LR99ES27, Faculty of Pharmacy, University of Monastir, 5000 Monastir, Tunisia; 3 Unit of Child Psychiatry, Monastir University Hospital, 5000 Monastir, Tunisia; 4 Laboratory of Antimicrobial Resistance LR99ES09, Faculty of Medicine of Tunis, University of Tunis El Manar, 1068 Tunis, Tunisia; 5 Diet, Microbiota, and Health Group, Instituto de Investigación Sanitaria del Principado de Asturias (ISPA), 33011 Oviedo, Spain

**Keywords:** Microbial communities, Microbiome, Microbiology

## Abstract

Alterations in gut microbiota and short chain fatty acids (SCFA) have been reported in autism spectrum disorder (ASD). We analysed the gut microbiota and fecal SCFA in Tunisian autistic children from 4 to 10 years, and results were compared to those obtained from a group of siblings (SIB) and children from the general population (GP). ASD patients presented different gut microbiota profiles compared to SIB and GP, with differences in the levels of *Bifidobacterium* and *Collinsella* occurring in younger children (4–7 years) and that tend to be attenuated at older ages (8–10 years). The lower abundance of *Bifidobacterium* is the key feature of the microbiota composition associated with severe autism. ASD patients presented significantly higher levels of propionic and valeric acids than GP at 4–7 years, but these differences disappeared at 8–10 years. To the best of our knowledge, this is the first study on the gut microbiota profile of Tunisian autistic children using a metataxonomic approach. This exploratory study reveals more pronounced gut microbiota alterations at early than at advanced ages in ASD. Although we did not account for multiple testing, our findings suggest that early interventions might mitigate gut disorders and cognitive and neurodevelopment impairment associated to ASD.

## Introduction

Autism spectrum disorder (ASD) is a neurodevelopmental condition characterized by impaired social interactions and communication, as well as restricted and repetitive patterns of behaviour, interest or activities^1^. The number of children with ASD has consistently risen in the last few years. According to a recent report, the global prevalence of autism was estimated to be 1% with preponderance among boys^2^. ASD is a multifactorial disorder involving a complex interaction between both genetic and environmental risk factors^3^. The genetic basis of ASD is heterogeneous and includes inherited and de novo gene mutations^4^. Accumulating evidence has indicated that other environmental elements, including exposure to pesticides, air pollution, dietary factors, medication, maternal infections and stress may play a role in the development of the disease^5^. In addition to the core symptoms of ASD, a wide range of gastrointestinal adverse symptoms are also frequently reported in autistic children, such as constipation, flatulence, diarrhoea and abdominal pain^6^.

Although the etiopathogenesis of ASD is not fully understood, recent findings have revealed alterations in the gut microbiota composition of these patients as compared to controls^7,8^. Gut microbiota harbours different commensal microorganisms including bacteria, fungi, archaea and viruses^9^. Gut communicates with the central nervous system through a complex bidirectional pathway, the so called “gut-brain-axis” which has been recently reported to play a major role in brain development and homeostasis^10^. In addition to the vagus nerve, the cross talk between gut microbes and the brain is facilitated through modulation of the immune system, the hypothalamic–pituitary–adrenal axis as well as via the production of microbial derived metabolites such as short chain fatty acids (SCFA)^11^.

SCFA, mainly acetic, butyric and propionic acids, are produced by intestinal microbial fermentation of undigested products from food components. These intestinal metabolites have crucial physiological effects including, maintenance of intestinal barrier and blood barrier integrity and modulation of the neuroinflammation process^12^. Notably altered fecal levels of SCFA have been found in ASD patients, suggesting alterations in the fermentation patterns of indigestible substrates in the gut^13,14^.

Although several studies have evaluated the gut microbiota composition and fecal levels of SCFA in individuals with ASD, data are still inconsistent and some of these studies have reported opposite results. In this regard, most authors reported a higher relative abundance of the phylum Bacteroidota in feces from children with ASD^15–17^, whereas some others did not corroborate these results^18,19^. Similarly, for the Bacillota phylum some studies showed higher levels in ASD^18–20^, others reported higher abundance of this phylum in samples from controls^16,17^ and others indicate no significant differences between ASD and controls groups^15,21^. It has been also found that ASD patients presented generally higher levels of the Beta-Proteobacteria class, and *Lactobacillus*, *Ruminococcus*, *Esherichia* and *Shigella* genera whereas the prevalence of *Bifidobacterium* and *Enterococcus* was reduced with respect to control individuals^18,22,23^. Along with gut microbiota composition, results from previous studies on fecal levels of SCFA were also sometimes contradictory. Thus, while Adams et al.^13^ reported lower fecal levels of these compounds in feces from children with ASD, other authors reported elevated levels^14,15,24^.

Additionally, the possible associations of the gut microbiota and its main metabolites with the infant´s age and the severity of ASD remain largely unexplored. The aim of the present study was to characterize the gut microbiota composition and the fecal levels of SCFA in Tunisian autistic children (ASD), as compared to their siblings (SIB) and to children from the general population (GP) matched by age and sex, and to determine the influence of age and severity of the disease on these traits.

## Results

### Gut microbiota diversity

**Figure 1:**
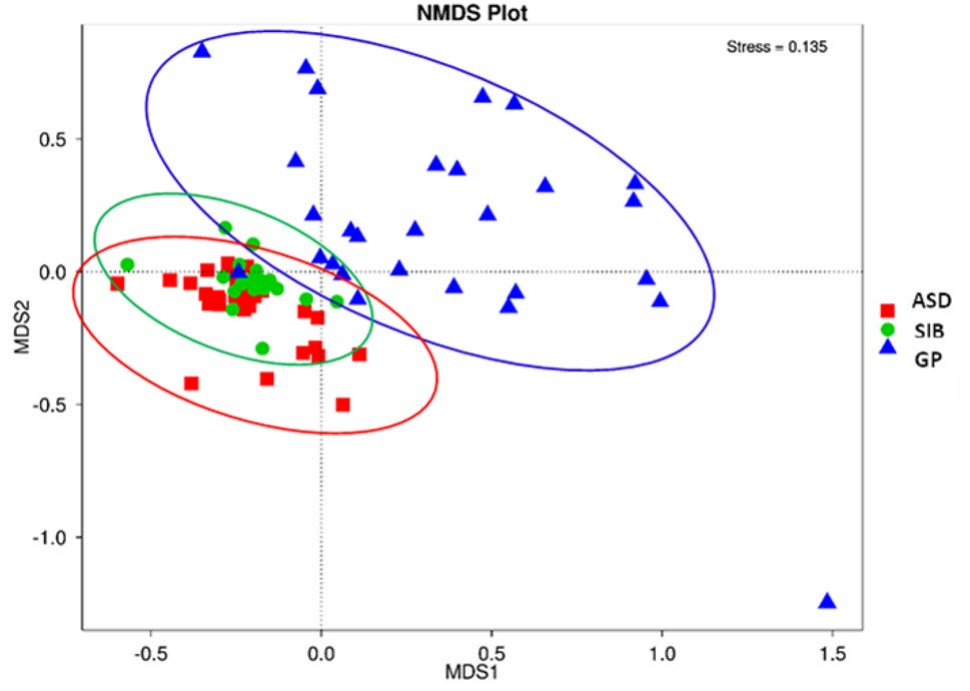
Beta diversity plot using Non-Metric Multi-Dimensional Scaling (NMDS) among fecal microbiota samples from the three groups of children: autistic (ASD), siblings (SIB) and children from the general population (GP) which are coloured in red, green and blue, respectively.

Beta diversity analysis was used to explore the dissimilarity among microbial communities from the fecal samples of the three groups of children considered in this work (Fig. 1). The non-multidimensial scaling (NMDS) analysis revealed that the gut microbiota of GP children clustered apart from that of ASD and SIB groups. The Adonis test indicated significant differences between ASD and both GP and SIB groups (*p* < 0.01 and *p* < 0.05 respectively), whereas no significant differences were found between GP and SIB.

**Figure 2:**
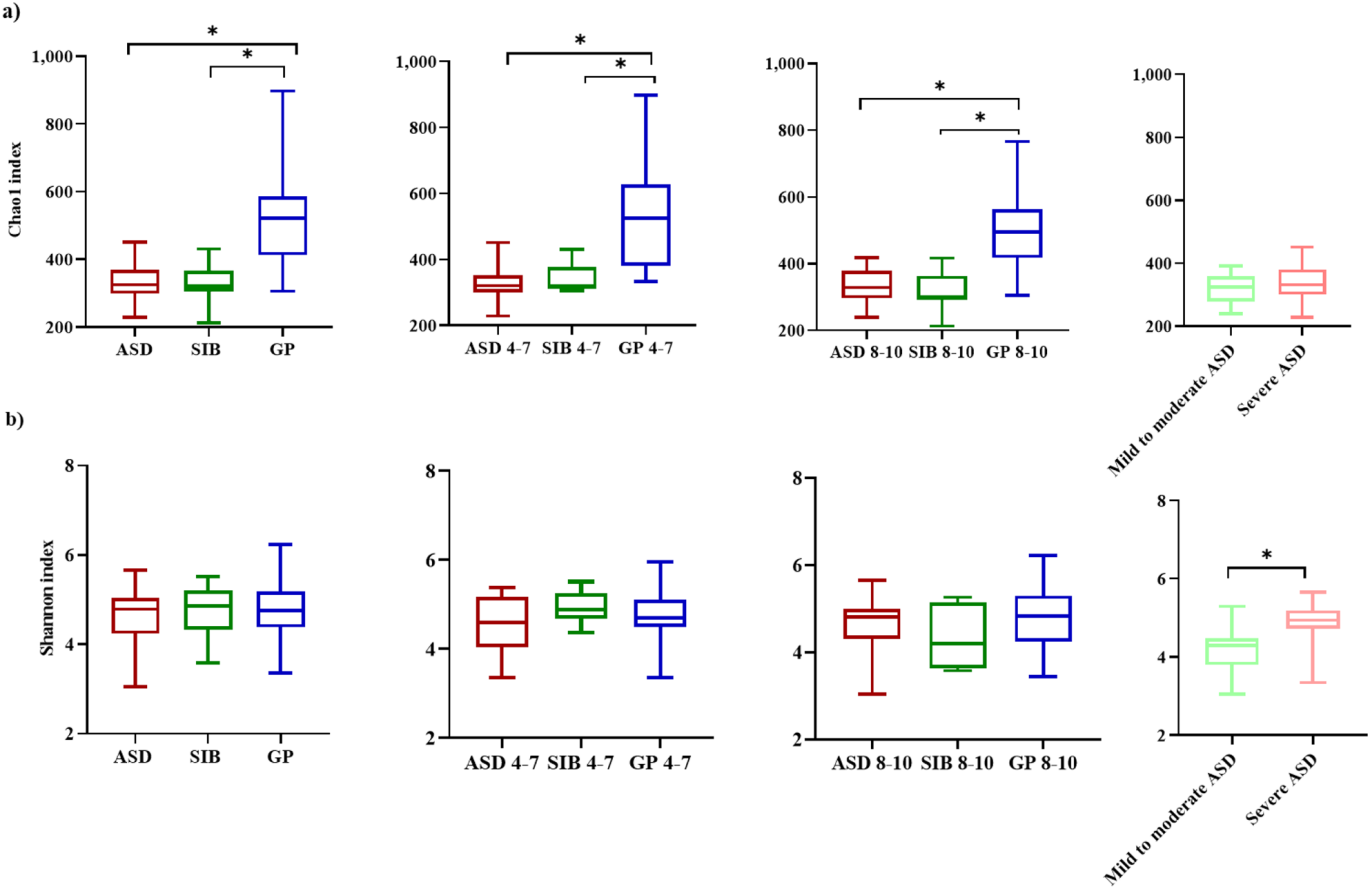
Alpha diversity: Chao1 (**a**) and Shannon (**b**) indices. Comparison among the three groups of children considered jointly, stratified by age (4–7 years and 8–10 years), and stratified by ASD severity (mild to moderate ASD or severe ASD). Box elements show the median and upper and lower quartiles. Statistically significant differences between groups with *p* < 0.05 are indicated with asterisk (*).

Chao1 (species richness) and Shannon (diversity of species) indices of alpha diversity were determined in ASD, SIB and GP considering the sample population as a whole, stratified by age and classified according to the severity of the disease (Fig. 2). The Chao1 index was significantly higher in fecal samples from the GP group than in ASD and SIB, independently of the range of age considered, whereas the Shannon index did not display differences. In contrast, comparison of alpha diversity according to the severity of the disease showed significantly higher values for the Shannon index in children with more severe symptoms as regards to those with mild to moderate ASD whereas no differences were found for the Chao1 index. Globally, these results indicate a similar diversity of species in GP, SIB and ASD groups, with a higher species richness in the GP group, whereas a higher diversity of species was found in severe ASD as compared to individuals with mild to moderate symptoms.

### Gut microbial composition evaluated by 16S rRNA gene profiling and qPCR

The metataxonomic analyses based on sequencing the V3–V4 region of the 16S rRNA gene, revealed substantial differences in composition among fecal microbiotas from the different children groups studied.

**Figure 3:**
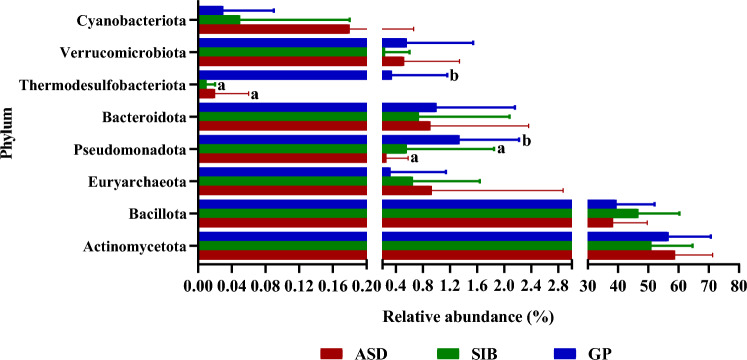
Relative abundance of the 8 phyla identified by sequencing the V3-V4 region of the 16S rRNA gene in fecal samples from autistic children (ASD), their siblings (SIB) and children from the general population (GP). Different letters on the bars (a, b) indicate significant differences among groups of children (*p* < 0.05).

**Figure 4:**
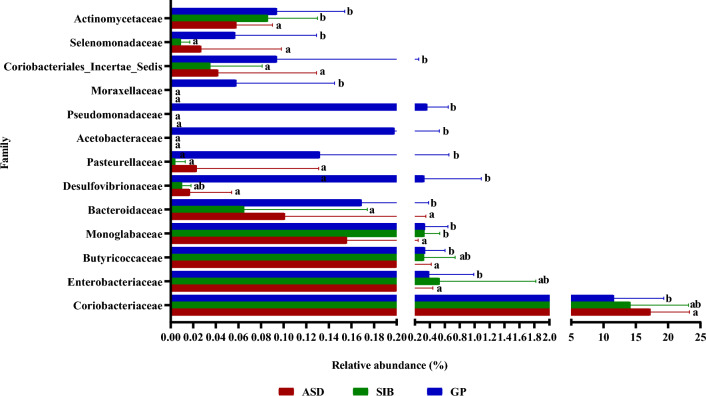
Relative abundance of bacterial families with significant differences among autistic children (ASD), their siblings (SIB) and children from the general population (GP). Different letters (a, b) indicate significant differences among groups of children for each family (*p* < 0.05).

Globally, the microbial communities of all samples from the three groups of children were assigned to 8 phyla, 40 families and 83 genera. At the phylum level, Actinomycetota and Bacillota were the two most abundant taxa in the gut microbiota of the three groups of children, followed by Bacteroidota, Pseudomonadota, Euryarchaeota, Verrucomicrobiota, Thermodesulfobacteriota and Cyanobacteriota (Fig. 3). The higher proportions of Actinomycetota and Bacillota in the fecal samples of the three groups of children were confirmed by qPCR (see Supplementary Fig. S1 online). Pseudomonadota and Thermodesulfobacteriota showed significantly greater relative abundances in feces from the GP (*p* < 0.001) children as compared to ASD and SIB groups (Fig. 3). Absolute levels of Actinomycetota were also significantly higher in samples from GP group compared to SIB (*p* < 0.05) (see Supplementary Fig. S1 online). At the family level, Bifidobacteriaceae and Coriobacteriaceae were the two most abundant in the gut of the three groups of children. We identified 13 bacterial families displaying significant differences in their relative abundance among GP, SIB and ASD groups (Fig. 4). Notably, Coriobacteriaceae was significantly more abundant in ASD than in GP group (18.22 ± 6.04 vs. 12.56 ± 7.75, *p* < 0.05) and in the SIB group (18.22 ± 6.04 vs. 15.09 ± 9.02, p = 0.056). In contrast other families, among them Pasteurellaceae, Bacteroidaceae, Acetobacteraceae, Pseudomonadaceae, Selenomonodaceae and Moraxellaceae, showed significantly higher proportions in the GP group than in ASD and SIB (Fig. 4). Interestingly, the Bifidobacteriaceae/Coriobacteriaceae ratio of relative abundances was significantly lower in the ASD group as compared to children belonging to GP group (see Supplementary Fig. S2 online). *Bifidobacterium* and *Collinsella* were the two most common bacterial genera in the gut of the three groups of children. Out of the 83 genera identified, 38 of them showed significant differences in their relative abundance among the groups of children (see Supplementary Table S1 online). Thus, significantly higher proportions of *Collinsella*, *Coriobacteriaceae UCG-003*, *Ruminococcus torques* group, *Sarcina*, *Slackia*, *Solobacterium*, *Methanosphera,* and *Lachnospiraceae NKA3A20-*group were found in ASD with respect to GP, with *Sarcina*, *Solobacterium* and *Methanosphera* also displaying higher relative abundances in ASD with respect to the SIB group. In contrast, the abundance of some genera, among others *Eggerthella*, *Ruminococcaceae*_*CAG-352*, *Klebsiella*, *Butyricicoccus* and *Citrobacter,* was significantly reduced in ASD children as compared to GP children. In particular, *Gluconobacter*, *Pseuodomonas*, *Kroppenstedtia*, *Acinetobacter* and *Megamonas* were only present in feces from the GP children (see Supplementary Table S1 online).

**Figure 5:**
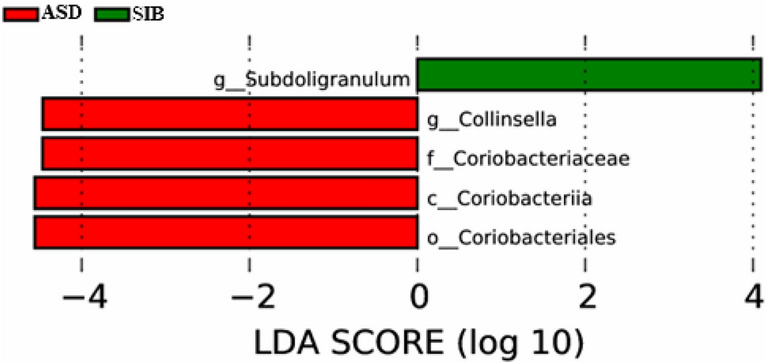
LDA scores for the microbial taxa differentially abundant among autistic children (ASD), their siblings (SIB) and children from the general population (GP) groups. Significantly discriminant bacterial groups were found only between ASD and SIB. Enriched bacterial taxa in ASD have positive LDA scores (red) and enriched bacterial taxa in SIB have negative LDA scores (green). Only the taxa with a *p* < 0.05 and LDA > 2.0 are shown.

We analyzed further those members of the bacterial community that could constitute differential markers among ASD, SIB and GP groups using to discriminate the effect size of each taxon differently represented in patients and controls^25^. The linear discriminant Effect Size (LEfSe) analysis performed among the three groups showed a differentially higher relative abundance of Coriobacteriaceae and *Collinsella* in the ASD group, and a differentially higher relative abundance of the *Subdoligranulum* genus in children from the SIB group (Fig. 5).

Therefore, differences in gut microbiota profiles occurred among the three groups of children considered, as well as between autistic children and children from the general population whereas specific microbial signatures differentiate autistic children from their siblings.

### Shifts on the gut microbiota composition as a function of age

To evaluate the influence of the infant’s age on the gut microbiota profiles, we separately compared the gut microbiota in children aged from 4 to 7 years and in those aged from 8 to 10 years.

**Figure 6:**
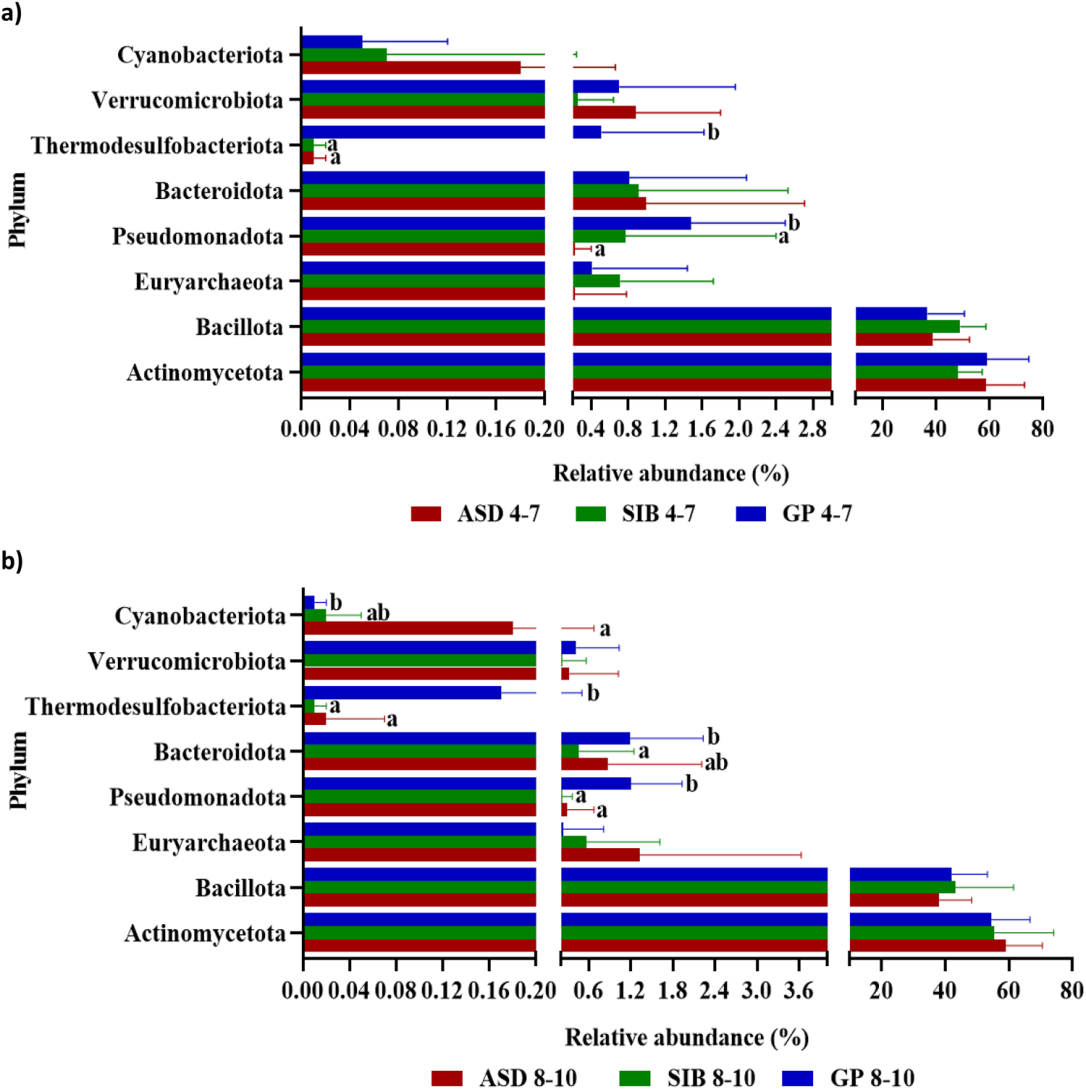
Relative abundance of the 8 phyla identified by sequencing the V3-V4 region of the 16S rRNA gene in fecal samples from autistic children (ASD), their siblings (SIB) and children from the general population (GP) stratified by age: (**a**) 4–7 years, (**b**) 8–10 years. Different letters on the bars (a, b) indicate significant differences among groups of children (*p* < 0.05).

**Figure 7:**
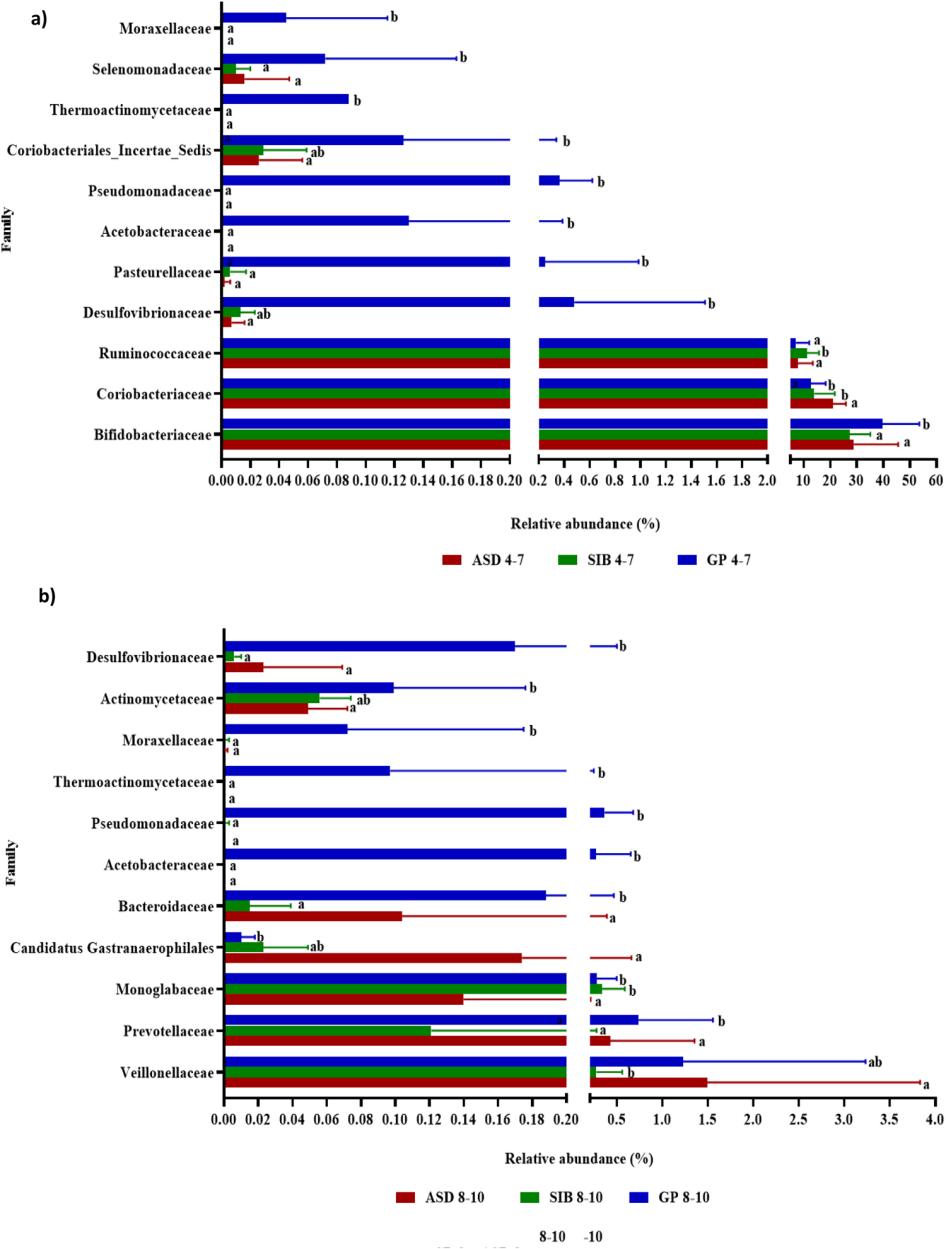
Relative abundance of bacterial families with significant differences among autistic children (ASD), their siblings (SIB) and children from the general population (GP), stratified by age: (**a**) 4–7 years, (**b**) 8–10 years. Different letters on the bars (**a**, **b**) indicate significant differences among groups of children for each family (*p* < 0.05).

At the age of 4–7 years, the phyla Pseudomonadota and Thermodesulfobacteriota were significantly more abundant in GP than in ASD and SIB groups (Fig. 6a). In addition, we found 11 bacterial families displaying significant differences in their relative abundance among the three groups of children (Fig. 7a). Among these, Bifidobacteriaceae were significantly more abundant in the GP group than in ASD and SIB, while Coriobacteriaceae were significantly more abundant in ASD than in GP and SIB (Fig. 7a). As a consequence, the Bifidobacteriaceae/Coriobactericeae ratio was higher in the GP group than in ASD (see Supplementary Fig. S2 online). Interestingly, the Ruminococaceae family was significantly more abundant in the SIB group than in GP and ASD. At the genus level, we identified 24 genera displaying significant differences between the three children groups (see Supplementary Table S2 online). Notably, *Bifidobacterium* displayed higher relative abundance in the GP group than in ASD and SIB whereas *Collinsella* was more abundant in the gut of the ASD children as compared to SIB and GP groups (see Supplementary Table S2 online).

At the age of 8–10 years, in addition to the significant differences in Pseudomonadota and Thermodesulfobacteriota already commented before at earlier ages, we found that the GP group presented a significantly higher abundance of Bacteroidota compared to SIB and lower abundance in Cyanobacteriota compared to the ASD group (Fig. 6b). Although no significant differences were found in the relative abundance of Coriobacteriaceae and Bifidobacteriaceae as occurred at 4–7 years, we identified 11 families with significant variations among groups (Fig. 7b). Moreover, in spite that the Bifidobacteriaceae/Coriobacteriaceae ratio remained considerably higher in the GP group than in children clustered in the ASD and SIB groups, these differences did not reach statistical significance, probably due to the high variability found in the GP group (see Supplementary Fig. S2 online). Remarkably, at the genus level no significant difference was found in the relative abundance of *Bifidobacterium* and *Collinsella,* as it occurred in younger children from 4 to 7 years of age, but notably 23 other minority genera displayed differences among the children’s groups (see Supplementary Table S3 online).

These results revealed differential alterations in the microbiota profile of ASD as a function of age. Differences found in the levels of *Bifidobacterium* and *Collinsella* in younger children tend to be attenuated at older ages.

### Differences on the gut microbiota composition associated to the severity of disease

**Figure 8:**
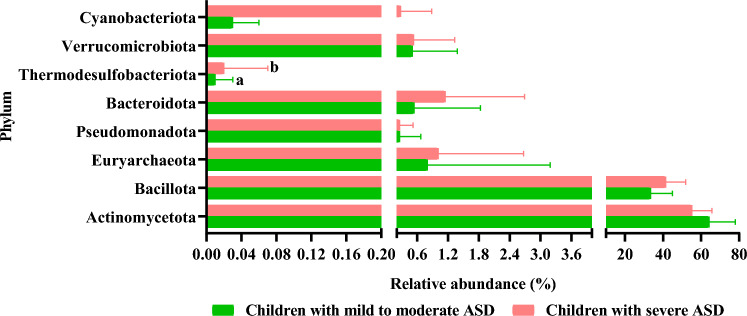
Relative abundance of the 8 phyla identified by sequencing the V3–V4 region of the 16S rRNA gene in fecal samples from autistic children’s subgroups: children with mild to moderate ASD and children with severe ASD. Different letters on the bars (**a**, **b**) indicate significant differences among groups of children (*p* < 0.05).

**Figure 9:**
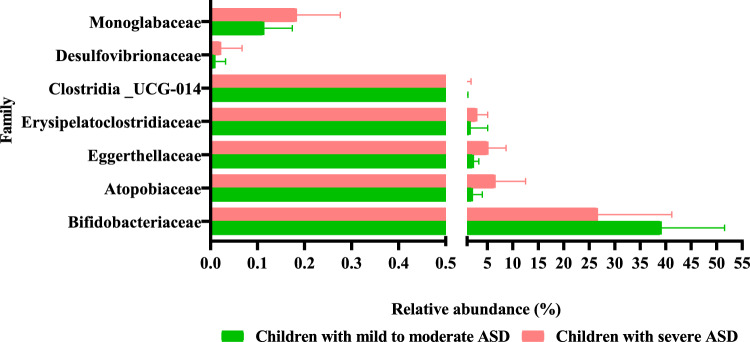
Relative abundance of bacterial families with significant differences (*p* < 0.05) between autistic children’s subgroups: children with mild to moderate ASD, children with severe ASD.

To understand whether the gut microbiota composition may be associated with the severity of autism, we performed comparisons between children with mild to moderate ASD and children with severe ASD, based on Childhood Autism Rating Scale^26^. Although globally the gut microbiota composition was similar between the two groups, some remarkable differences were found. At the phylum level, only Thermodesulfobacteriota displayed significantly higher abundance in children with severe ASD than in children with mild to moderate ASD (Fig. 8). We identified 7 families with significant differences between the two subsets; thus, the relative abundance of Bifidobacteriaceae was significantly lower in children with severe ASD than in children with mild to moderate ASD, whereas the remaining families displayed significantly lower relative abundance in children with mild to moderate ASD than in those with severe symptoms (Fig. 9). The Bifidobacteriaceae/Coriobacteriaceae ratio was higher in children with mild to moderate ASD than in those with severe ASD, without reaching statistical significance (*p* > 0.05) (see Supplementary Fig. S2 online). Eight genera showed significant differences between the two groups, with *Bifidobacterium* being reduced in children with severe ASD symptoms in contrast with the other 7 genera that were more common in severe autists (see Supplementary Table S4 online).

Our results point to differences in the microbiota of autistic children as related to the severity of the disease and evidenced the lower abundance of the genus *Bifidobacterium* as one of the main traits of the microbiota composition related with the severity of autism.

### Fecal SCFA profiles

Acetic acid was the most abundant compound detected in fecal samples followed by propionic acid and then butyric acid, with minor amounts of branched chain fatty acids (BCFA: isobutyric and isovaleric acids) and other minor SCFA. No significant differences were found for any of these compounds among ASD, SIB and GP groups, considering jointly the whole sample at all ages. However, when comparisons were made stratifying by age, significantly higher levels of propionic and valeric acids as well as the sum of minor SCFA (isobutyric + isovaleric + valeric + caproic acids) were found in ASD at the age 4–7 years, but these differences disappeared at the age of 8–10 years (Table 1). These results evidenced alterations in SCFA profiles between ASD and GP children in younger individuals that are attenuated at older ages.

**Table 1:** Concentrations of fecal SCFA in autistic children (ASD), their siblings (SIB) and children from the general population (GP) in the whole sample and stratified by age (4–7 years and 8–10 years).

				Children between 4 and 7 years			Children between 8 and 10 years		
	ASD	SIB	GP	ASD 4–7	SIB 4–7	GP 4–7	ASD 8–10	SIB 8–10	GP 8–10
Acetic (mM)	101.709 ± 27.425	95.109 ± 25.336	101.458 ± 28.168	108.562 ± 28.055	98.624 ± 28.009	102.514 ± 26.965	97.902 ± 27.110	89.585 ± 21.284	100.401 ± 30.305
Propionic (mM)	39.036 ± 15.281	34.698 ± 13.568	32.942 ± 13.566	43.563 ± 15.648^a^	37.525 ± 11.921^ab^	28.197 ± 8.530^b^	36.520 ± 14.915	30.256 ± 15.718	37.687 ± 16.155
Butyric (mM)	30.614 ± 11.645	30.444 ± 13.078	31.450 ± 13.017	32.982 ± 12.951	32.090 ± 15.272	33.512 ± 12.865	29.298 ± 11.021	27.858 ± 9.118	29.388 ± 13.315
Isobutyric (mM)	4.276 ± 2.425	3.451 ± 1.502	3.564 ± 1.803	5.180 ± 2.012	3.546 ± 1.651	3.410 ± 1.724	3.775 ± 2.540	3.300 ± 1.343	3.719 ± 1.932
Isovaleric (mM)	6.692 ± 4.107	5.311 ± 2.367	5.475 ± 3.103	8.059 ± 3.560	5.407 ± 2.561	5.204 ± 2.854	5.933 ± 4.286	5.161 ± 2.215	5.745 ± 3.420
Valeric (mM)	5.205 ± 2.858	4.040 ± 2.104	4.320 ± 2.371	6.507 ± 2.907^a^	4.543 ± 2.404^ab^	3.921 ± 2.130^b^	4.482 ± 2.637	3.249 ± 1.309	4.719 ± 2.606
Caproic (mM)	1.578 ± 1.252	1.381 ± 1.039	1.598 ± 1.534	1.786 ± 1.428	1.514 ± 1.135	1.752 ± 1.862	1.462 ± 1.171	1.171 ± 0.908	1.444 ± 1.171
Total SCFA (mM)	189.113 ± 52.174	174.436 ± 47.581	180.809 ± 46.848	206.642 ± 54.317	183.252 ± 51.523	178.513 ± 38.547	179.376 ± 49.803	160.582 ± 40.296	183.105 ± 55.327
BCFA (mM)	10.969 ± 6.520	8.762 ± 3.864	9.039 ± 4.899	13.239 ± 5.558	8.953 ± 4.209	8.614 ± 4.570	9.708 ± 6.816	8.462 ± 3.549	9.465 ± 5.345
Minor SCFA (mM)	17.753 ± 9.004	14.184 ± 5.992	14.958 ± 7.122	21.533 ± 7.437^a^	15.012 ± 6.630^ab^	14.289 ± 6.876^b^	15.654 ± 9.296	12.882 ± 5.023	15.628 ± 7.556

Results are presented as mean values ± standard deviation. Total SCFA: acetic + propionic + butyric + isobutyric + isovaleric + valeric + caproic acids. BCFA: isovaleric + isobutyric. Minor SCFA: isovaleric + isobutyric + valeric + caproic acids. Different letters (a, b) indicate significant differences among groups (*p* < 0.05).

No significant differences were either found between ASD children with mild to moderate and severe symptoms of the disease in the absolute levels of SCFA (Table 2). However, molar proportions of isobutyric and isovaleric BCFA were significantly higher (*p* < 0.05) in individuals with severe ASD as regards to those with mild to moderate ASD (2.714 ± 1.227 vs. 1.775 ± 0.867 for isobutyric; 4.404 ± 2.163 vs. 2.673 ± 1.561 for isovaleric, respectively).

**Table 2:** Concentrations of fecal SCFA between autistic children’s subgroups: children with mild to moderate ASD and children with severe ASD. Results are presented as mean values ± standard deviation.

	ASD children’s subgroups	
	Mild to moderate ASD	Severe ASD
Acetic (mM)	107.707 ± 19.393	97.828 ± 31.519
Propionic (mM)	38.253 ± 10.543	39.542 ± 17.996
Butyric (mM)	31.460 ± 7.410	30.066 ± 13.918
Isobutyric (mM)	3.349 ± 1.611	4.877 ± 2.707
Isovaleric (mM)	4.998 ± 2.845	7.789 ± 4.489
Valeric (mM)	4.451 ± 3.302	5.693 ± 2.516
Caproic (mM)	1.552 ± 1.469	1.595 ± 1.139
Total SCFA (mM)	191.773 ± 30.610	187.392 ± 63.245
BCFA (mM)	8.347 ± 4.436	12.666 ± 7.188
Minor SCFA (mM)	14.352 ± 7.290	19.954 ± 9.513

Total SCFA: acetic + propionic + butyric + isobutyric + isovaleric + valeric + caproic acids. BCFA: isovaleric + isobutyric acids. Minor SCFA: isovaleric + isobutyric + valeric + caproic acids.

### Association of the gut microbiota and SCFA profiles

**Figure 10:**
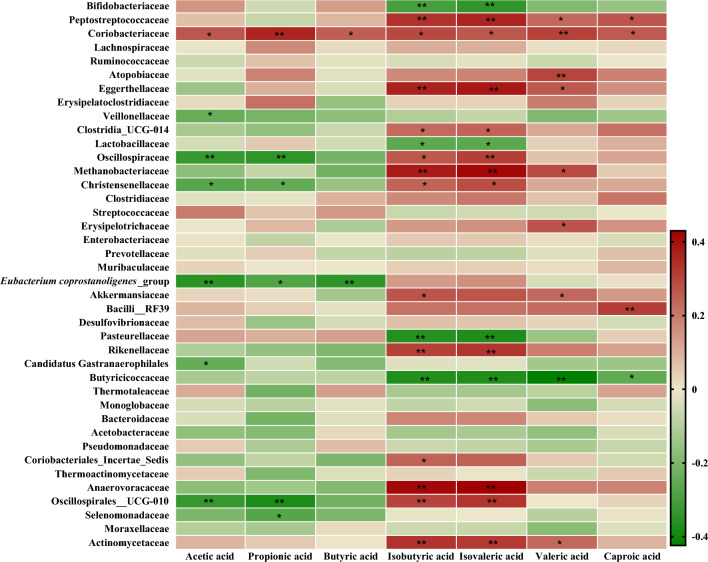
Heatmap showing Spearman correlations between SCFA profile (columns) and gut bacterial families (rows). Green and red colors denote negative and positive associations, respectively. The intensity of colors represents the degree of association between variables. Asterisks indicate significant associations: *p < 0.05 and **p < 0.01.

To further explore possible associations between the gut microbiota and SCFA in autistic and control groups, Spearman correlations were performed between fecal levels of SCFA and the different microbial groups identified by 16S rRNA gene profiling at the family level (Fig. 10).

Coriobacteriaceae, microorganisms significantly increased in the ASD group, correlated positively with all SCFA analyzed (*p* < 0.05). In contrast, Oscillospiraceae (rs = − 0.340, *p* = 0.003), Christensenellaceae (rs = − 0.246, *p* = 0.035), *Eubacterium coprostanoligenes*_group (rs = − 0.280, *p* = 0.016), Oscillospirales__UCG-010 (rs = − 0.375, *p* = 0.001) and Selenomonadaceae (rs = − 0.272, *p* = 0.019), generally enriched in SIB and GP groups were negatively correlated with propionic acid.

Acetic acid correlated negatively with Veillonellaceae (rs = − 0.240, *p* = 0.039), Oscillospiraceae (rs = − 0.323, *p* = 0.005), Christensenellaceae (rs = − 0.268, *p* = 0.021), *Eubacterium coprostanoligenes*_group (rs = − 0.348, *p* = 0.002), Candidatus Gastranaerophilales (rs = − 0.243, *p* = 0.037) and Oscillospirales_UCG-010 (rs = − 0.330, *p* = 0.004), whereas butyric acid was negatively associated with *Eubacterium coprostanoligenes*_group (rs = − 0.340, *p* = 0.003).

The branched chain fatty acids isobutyric and isovaleric were positively associated with Peptostreptococaceae, Eggerthellaceae, Oscillospiraceae, Methanobacteriaceae, Christensenellaceae, Akkermansiaceae, Clostridia_UCG-014, Rikenellaceae, Anaerovoracaceae, Oscillospirales_UCG-010 and Actinomycetaceae and negatively correlated with Bifidobacteriaceae, Lactobacillaceae, Pasteurellaceae, and Butyricocaceae (*p* < 0.05).

Valeric acid was positively correlated with Atopobiaceae (rs = 0.300, *p* = 0.009), Peptostreptococaceae (rs = 0.233, *p* = 0.046), Eggerthellaceae (rs = 0.262, *p* = 0.024), Methanobacteriaceae (rs = 0.290, *p* = 0.012), Erysipelotrichaceae (rs = 0.272, *p* = 0.019), Akkermansiaceae (rs = 0.232, *p* = 0.047) and Actinomycetaceae (rs = 0.233, *p* = 0.045) and negatively correlated with Butyricicocaceae (rs = − 0.424, *p* = 0.000). Caproic acid correlated positively with Peptostreptococcaceae (rs = 0.270, *p* = 0.020) and Bacilli_RF39 (rs = 0.314, *p* = 0.007) and negatively with Butyricicocaceae (rs = − 0.253, *p* = 0.030).

## Discussion

The high prevalence of gastrointestinal (GI) disorders in autistic patients suggests the involvement of the gut microbiota in the etiopathogenesis of ASD. Although numerous studies have found abnormalities in gut microbiota and levels of SCFA, few of them have explored alterations of the gut microbiota and its metabolic activity as related with age and severity of the disease. Thus, in the present study, we analyzed the gut microbiota composition and fecal levels of SCFA, the main products from the microbiota fermentation activity in the colon, in Tunisian autistic children, as compared to their neurotypical brothers and sisters and to children from the general population and we investigated the effect of age and severity of the disease on these features. To the best of our knowledge this is the first report using metataxonomic approaches for the study of the gut microbiota in a Tunisian children population at pre-school and school-ages.

Metataxonomic analyses carried out in the present work revealed important alterations in the gut microbiota of children with ASD as compared with their SIB and GP. Beta diversity analysis clustered separately the gut microbiota of the GP group from that of ASD and SIB, but did not separate these last two groups. Nevertheless, the Adonis test revealed significant differences between ASD and both SIB and GP, but not between the two neurotypical control groups, SIB and GP. Our results are in accordance with previous studies showing clear differences between autistic and neurotypical controls^15,18,27,28^, differences being less evident when comparing ASD subjects and their neurotypical siblings^27,28^. These findings are probably reflecting important alterations in the gut microbial communities of ASD, and a strong family environmental influence as well in the gut microbiota of ASD and their SIB. In this respect, it is well known the influence of the surrounding environment in the development of the gut microbiota, which is especially important at early ages of life^29^. Concerning alpha diversity, we did not find differences using the Shannon index in the gut microbiota of the three groups of children. However, Chao1 index which reflects the species richness in the gut microbiota, was higher in GP than in ASD and SIB. These results could be reflecting higher diversity of minor and rare species among individuals from the GP group in contrast to a more uniform microbiota in the groups of ASD and SIB. In this respect, some studies found similar alpha diversity in ASD and controls^30–32^ whereas others reported significant differences between these two groups. Thus, De Angelis et al.^24^ and Cao et al.^7^ found greater microbial diversity in subjects with ASD than in controls whereas Kang et al.^33^ and Liu et al.^17^ found reduced microbial diversity in the gut of children with ASD. Higher species diversity in neurotypical individuals with respect to autists using different indices has been reported in previous studies^16,33^. Apart differences on gut microbiota parameters highlighted by the different indices to determine diversity, some inconsistencies among different studies could be partly explained by the heterogeneity among studies in the populations analysed and the different techniques used to collect and process data, i.e. differences in diagnostic criteria (e.g., ADOS, ADI-R, CARS), methods for microbiota analysis (16S rRNA sequencing, qPCR, culture), as well as differences in genetic background, environmental factor as diet, and anthropometric factors as age^16^.

Numerous studies have reported shifts in the composition of the gut microbiota in ASD children as compared to neurotypical individuals^16–18,30,34^. Here, in our study, we investigated the gut microbial community at different taxonomic levels (phylum, family and genus). In contrast to previous reports^17,19,28,33^, we found that in our cohort Actinomycetota was the most abundant microbial phylum in the gut of ASD, SIB and GP, without significant differences among the three groups. Higher abundance of Bacillota in children with ASD and neurotypical controls has been previously reported^17,19,24^. In our case, the predominance of Actinomycetota could be related with environmental and anthropometric factors as dietary patterns and/or age of individuals, as well as with the DNA sequencing technique and primers used. We also found that relative abundances of Pseudomonadota and Thermodesulfobacteriota were significantly higher in GP children as compared to ASD and SIB, in agreement with the higher abundance of Pseudomonadota recently reported by Zou et al. in neurotypical subjects as compared to autists^35^.

At the taxonomic family level, we found Coriobacteriaceae and Bifidobacteriaceae as the two most abundant in our cohort. More specifically, Coriobacteriaceae were significantly more abundant in ASD than in GP, consistent with the study by Ding et al.^28^. Coriobacteriaceae play important roles in bile salt formation, steroid conversion and activation of dietary polyphenols; however, they can be also considered pathobionts as they have been linked to a number of extraintestinal infections such as bacteraemia, periodontitis and vaginosis^36,37^. Therefore, their presence at increased levels may be potentially harmful in ASD patients. In contrast, Bifidobacteriaceae were more enriched in the gut microbiota of GP than in ASD, consistent with the results of Coretti et al.^15^. In addition, other families mainly belonging to the Bacillota and Pseudomonadota phyla, such as Pasteurellaceae, Acetobacteraceae, Pseudomonadaceae, Selenomonodaceae and Moraxellaceae were less abundant in ASD and their SIB, as compared to GP.

*Collinsella* and *Bifidobacterium* were the two most abundant genera found by us in the gut of the three groups of children. Our results showed that relative abundances of *Collinsella*, *Coriobacteriaceae UCG-003*, *Ruminococcus torques* group, *Sarcina*, *Solobacterium*, *Methanosphera* and *Lachnospiraceae NKA3A20-*group were significantly more abundant in children with ASD. Increased levels of *Collinsella* in ASD subjects were in line with the results obtained by other authors in previous studies^18,28^. *Collinsella* has been reported to be involved in a variety of diseases^38^. Elevated levels of *Collinsella* have been found in patients with type 2 diabetes mellitus^39^. In overweight and obese pregnant women, the abundance of *Collinsella* was associated with high levels of insulin, triglycerides, and also with very low-density lipoproteins (VLDL) and low fibre intake^40,41^. In healthy individuals, elevated concentrations of *Collinsella* have been related to increased levels of total cholesterol and LDL cholesterol, clearly showing the influence of *Collinsella* in the host metabolism^42^. The higher abundance of *Ruminococcus torques* group found in stools of our ASD children were consistent with previous studies^15,43^. This genus, known as a mucin degrading bacteria, has been linked to impaired gut barrier integrity, which may be contributing to the development of the “leaky gut” described in ASD children. A “leaky gut” allows bacteria and/or bacterial-derived metabolites to enter the systemic circulation, where they can cross the Blood–Brain Barrier (BBB) and cause neuronal dysfunction and inflammation. Notably, in our study some genera, mainly from the phylum Pseudomonadota, were only found in GP children, such as *Gluconobacter*, *Pseuodomonas*, *Kroppenstedtia*, *Acinetobacter* and *Megamonas*. In fact, Pseudomonadota are believed to play a crucial role in preparing the gut for colonization by the strict anaerobes required for healthy gut function by consuming oxygen and reducing the redox potential in the gut environment^44^. The presence of these genera only in the group of GP is in good agreement with the higher values of the Chao1 index of alpha diversity obtained for this group.

Diet, mode of birth, and other factors could affect gut microbiota composition^45^ and there is some scientific evidence indicating that children with ASD tend to have a dietary pattern characterized by high fat and low vegetable and fruits intake^46,47^. However, we cannot discern in our sample to which extent shifts in the gut microbiota between ASD and their SIB could be attributed to dietary and anthropometric and lifestyle factors or to endogenous differences caused by the disease itself. In spite of this and beyond the similarities and differences existing between the gut microbiota of ASD children and their SIB, LefSe analysis conducted by us revealed specific bacterial taxa representing gut microbial signatures that differentiates one group from another. ASD children showed a differentially higher relative abundance of Coriobacteriaceae and *Collinsella* whereas the differentially abundance of the genus *Subdoligranulum*, from the Bacillota phylum, was characteristic of the gut microbiota from the SIB group. Remarkably, the genus *Subdoligranulum*, a butyrate producer member of the Ruminococcaceae family has been positively correlated in humans with a healthy metabolic status, although causality has not been proven to present^48^.

The gut microbiota can produce different types of metabolites, including SCFA through fermentation^49^. Several human and animal studies have shown that SCFA play a crucial role in metabolic homeostasis and overall gut health. SCFA (primarily acetic, butyric, and propionic acids) are sources of energy for enterocytes and have been shown to improve gut barrier integrity, glucose and lipid homeostasis, regulate immune function and the inflammatory response^50^. In our study, considering the whole human sample, fecal SCFA levels did not differ significantly between ASD children and healthy subjects (SIB and GP), as previously reported^27^. However, other studies on SCFA and ASD reported contradictory results, fecal levels of SCFA being either lower^13,24^ or higher^14,15^ in children with autism with respect to neurotypical individuals. This inconsistency among studies could be due to differences in populations analyzed, fecal sample processing and analytic methods, and diets from volunteers. In spite of this, we found in our cohort a positive correlation between the relative abundance of the Coriobacteriaceae family which was significantly more abundant in feces from ASD, and levels of fecal SCFA, mainly propionic and valeric acids (strong positive correlation). Some SCFA have shown effects on the gut-brain axis communication^51^. It has been reported that propionic acid modulate in vitro human neural stem cells, inducing gliosis and neuroinflammation, symptoms that are commonly described in ASD^52^. Moreover, intracerebroventricular and intraventricular administration of propionic acid in animal models induce autistic-like behaviours such as hyperactivity, repetitive behaviours and social communication impairment^53–55^.

One of the most important factors explaining differences in the gut microbial community between individuals is age^56^. Several studies have revealed age-related changes in the human gut microbiota^57^. In this way, higher levels of Enterobacteria and *Bifidobacterium* have been reported in children than in adults^58,59^. In contrast, *Bacteroides*, *Eubacterium* and Clostridiaceae have been found at lower levels in children compared to subjects with more advanced age^56^. These age-related changes in the gut microbiota could be also reflected in changes on SCFA levels in the gut as related to age. Thus, here we have analyzed differences in gut microbiota and fecal SCFA by stratifying our sample separately into two ranges of age: 4–7 years and 8–10 years. In the two ranges of age considered, we found that the Chao1 index was significantly higher in stool samples from the GP group than in ASD and SIB, while the Shannon index showed no differences, indicating that there is no age-related changes in alpha diversity of the gut microbiota in our cohort, which is in contrast with a recent study showing that alpha diversity increased with age in GP children as compared to ASD children^60^. In spite of this, in our cohort the three groups of children showed differential gut microbial profiles at the two ranges of age at phylum, family and genus levels. Interestingly, at the age of 4–7 years, Bifidobacteriaceae family and *Bifidobacterium* genus were significantly more abundant in the GP group than in ASD and SIB while Coriobacteriaceae and *Collinsella* were significantly higher in the ASD group than in SIB and GP and these differences disappeared at the age 8–10 years. In a similar way, at the age 4–7 years, ASD children displayed significantly increased fecal levels of propionic and valeric acids than GP children and these differences attenuated with age. Families Bifidobacteriaceae and Coriobacteriaceae, are generally more abundant in early childhood and their abundance decrease significantly with age^61,62^. There are currently no studies available on age-dependent changes in fecal SCFA profiles in autism although it seems that levels of acetic and propionic acids tend to decrease along life^63^. Moreover, we recently reported in the same population of ASD children a more altered fecal amino acids profile in younger than in the older individuals^64^. Our results clearly indicate that differences occurring in the microbiota composition and some fecal metabolites in autistic children at early childhood tend to attenuate with age. However, it is possible that these early alterations could exert a more durable impact in the gut and neuro-endocrine-immune system through the gut-brain axis, imprinting a long-lasting effect on physiology and health. Therefore, early detection and diagnosis of ASD is crucial in order to carry out interventions at initial stages that could contribute to mitigate the long-term cognitive and digestive alterations occurring in this disease.

Children with severe symptoms seem to display a different microbiota profile compared to children with mild to moderate ASD. We found greater diversity of species in severe ASD compared to children with mild to moderate symptoms. Moreover, Thermodesulfobacteriota was more abundant in children with severe ASD, whereas Bifidobacteriaceae and *Bifidobacterium* abundances were higher in children with mild to moderate ASD. This finding supports previous studies reporting changes in gut microbiota based on ASD severity^23,28,34^. The lower abundance of *Bifidobacterium* in children with severe ASD represents a key feature of microbiota composition related to the severity of ASD. Although other published studies, also support alterations in intestinal microbiota according to ASD severity, some authors reported no differences^20,65^. To this regard the different scales used in studies for measuring the severity of the disease may support differences in the conclusions obtained.

This study has potential limitations as not including the evaluation of dietary habits, the absence of analysis of the microbiota in other parts of the gastrointestinal tract as mouth and stomach and the relatively low sample size. On the other hand the exploratory nature of our work, in which multiple testing was not performed precludes to extract conclusions about the possible association among variables and the influence of such possible associations on the gut microbiota. In spite of that, our work revealed that the gut microbiota of ASD children differed from that of non autistic controls. We report here for the first time age- and disease severity-associated changes in the gut microbiota of ASD children in Tunisia, and provide the first study on gut microbiota at pre-school and school ages in this country by high-throughput sequencing technologies. Alterations in gut microbiota and SCFA profiles are more pronounced at early age and tend to mitigate in late childhood. This points to early interventions that could counteract at least partly the important damage caused by this disease on gut disorders and on the cognitive function and neurodevelopment. The aberrant fecal microbiota and its metabolites may play an important role in the pathogenesis of the gut-brain-axis in ASD patients. Larger ASD cohorts studies involving microbiota patterns, dietary assessment, and inflammation pathways are needed to confirm the link between gut microbiota- derived metabolites and the central nervous system.

## Materials and methods

### Subjects

This is an exploratory study in which 74 Tunisian children aged from 4 to 10 years (57 boysand 17 girls) were enrolled for this study between January 2019 and February 2020. ASD children were recruited at the Child and Adolescent Psychiatry Unit-Department of Psychiatry, Fattouma Bourguiba University Hospital, Monastir whereas unrelated controls from the general population were recruited from kindergartens and schools at the city of Monastir, Tunisia. Participants were clustered into three groups: 28 ASD (22 boys and 6 girls; with a mean age of 7.93 ± 2.05 years), 18 age matched SIB (brothers and sisters of autistic children, 5 boys and 13 girls; with a mean age of 7.56 ± 1.89 years) and 28 age- and sex-matched unrelated children from GP (22 boys and 6 girls; with a mean age of 7.29 ± 2.09 years). ASD diagnosis was carried out according to the Diagnostic and Statistical Manual for Mental Disorders (DSM-5)^66^, the Autism Diagnostic Inventory-Revised (ADI-R)^67^, and the Autism Diagnostic Observation Schedule-2 (ADOS-2)^68^ by an expert and trained clinician. The severity of ASD was assessed using the Childhood Autism Rating Scale (CARS) dividing autistic children into two subgroups: 11 autistic with mild to moderate ASD (CARS score ranged between 30 and 36), 17 autistic with severe ASD (CARS score ranged between 37 and 60)^26^. The inclusion criteria of individuals in the ASD group comprised of children whose ages ranged between 4 and 10 years and who had been clinically diagnosed with ASD using internationally recognized and validated tools, namely DSM-5, ADOS-2, ADI-R, and CARS. Similarly, for the SIB and GP groups, we included children who were in the same range of age as the ASD group but who did not exhibit any signs of developmental disorders or psychiatric illnesses. The exclusion criteria for all the participants from the three groups of children included: having infections, other neurological disorders not strictly related to autism, type 1 diabetes, genetic syndromes, unbalanced or special diets, celiac disease, food intolerance, or inflammatory bowel disease. Subjects in this study were not treated with antibiotics or antifungals, and had not taken probiotics and/or prebiotics for at least 1 month prior to the sampling process. We stratified the three groups of children (ASD, SIB, GP) into two ranges of age: children aged between 4 and 7 years (n = 35, 22 boys and 13 girls; with a mean age of 5.74 ± 1.48 years) and children aged between 8 and 10 years (n = 39, 27 boys and 12 girls; with a mean age of 9.05 ± 1.68 years), considering that most autistic children in Tunisia remain at home until the age of 7 years (influence of family environment, mainly) whereas from the age of 8 years most of them are institutionalized or assist to specialized centers (predominant influence of external factors over the family environment). Demographic and clinical characteristics of the participants have been described previously in detail^64^. We also evaluated the frequency of GI symptoms in the three groups of children (ASD, SIB, GP) as such, stratified by age (4–7 years, 8–10 years) and according to the severity of ASD (children with mild to moderate ASD, children with severe ASD) (Table 3). The GI symptoms assessed included constipation, diarrhea, abdominal pain, oesophageal reflux and vomiting.

**Table 3:** Number and frequency of GI symptoms in autistic children (ASD), siblings (SIB) and children from the general population (GP) as a whole sample, stratified by age (4–7 years, 8–10 years) and according to the severity of ASD (mild to moderate ASD, severe ASD).

	Whole 3 groups			Children aged between 4 and 7 years			Children aged between 8 and 10 years			Autistic children’s subgroups	
	ASD	SIB	GP	ASD 4–7	SIB 4–7	GP 4–7	ASD 8–10	SIB 8–10	GP 8–10	Mild to moderate ASD	Severe ASD
GI symptoms n (%)	18 (64.3)	8 (44.4)	13 (46.4)	7 (70)	6 (54.5)	5 (35.7)	11 (61.1)	2 (28.6)	8 (57.1)	4 (36.4)	14 (82.4)
Constipation	17 (60.7)	3 (16.7)	5 (17.9)	7 (70)	3 (27.3)	3 (21.4)	10 (55.6)	0 (0)	2 (14.3)	3 (27.3)	14 (82.4)
Diarrhea	1 (3.6)	1 (5.6)	0 (0)	0 (0)	0 (0)	0 (0)	1 (5.6)	1 (14.3)	0 (0)	1 (9.1)	0 (0)
Vomiting	1 (3.6)	0 (0)	0 (0)	0 (0)	0 (0)	0 (0)	1 (5.6)	0 (0)	0 (0)	0 (0)	1 (5.9)
Oesophageal reflux	1 (3.6)	0 (0)	0 (0)	0 (0)	0 (0)	0 (0)	1 (5.6)	0 (0)	0 (0)	1 (9.1)	0 (0)
Abdominal pain	7 (25)	4 (22.2)	10 (35.7)	2 (20)	3 (27.3)	4 (28.6)	5 (27.8)	1 (14.3)	6 (42.9)	3 (27,3)	4 (23.5)

This study was conducted in accordance with the fundamental principles of the Declaration of Helsinki and was approved by the Ethics Committee of the Faculty of Medicine of Monastir (reference IORG 0,009,738 N°18/OMB 0990-0279) and by the Bioethics Committee of CSIC (reference 172/2020). Written informed consent was obtained from the parents and/or legal guardians of the enrolled subjects.

### Fecal sample collection, transportation and processing

Fecal samples from all participants were collected in sterile containers and transported to the Laboratory of Transmissible Diseases and Biologically Active Substances at the Faculty of Pharmacy in Monastir (Tunisia) within 1 h after collection. All samples were immediately frozen at − 20 °C. Frozen feces were imported on dry ice by the laboratory of IPLA-CSIC (Spain), following the requirements and rules for international packaging and transportation of biological substances. Prior to analysis, fecal samples were thawed, diluted 1/10 (w/v) in phosphate buffered saline (PBS), homogenized in a Lab-Blender 400 stomacher (Seward Medical, London, UK) for 5 min and centrifuged at max rpm for 15 min. The resulting cell-free supernatants and cell pellets were stored separately at − 20 °C until further analysis.

### Bacterial DNA isolation

Total bacterial DNA isolation from cell pellets was performed following Q Protocol for DNA extraction of fecal samples defined by the International Human Microbiome Standards Consortium^69^. Isolated DNA was kept frozen at − 20 °C until further analysis.

### Analysis of fecal microbiota by 16S rRNA gene profiling

The isolated DNA was used as template for partial sequencing (hypervariable V3–V4 region) of the 16S rRNA gene on the Illumina platform (NovaSeq 6000) using the pair primers 341F (CCTAYGGGRBGCASCAG) and 806R (GGACTACNNGGGTATCTAAT) following in-house protocols (Novogene, Beijing, China). Based on samples’ unique barcode, raw 250 bp paired-end reads were assigned to different samples and merged to raw tags by using FLASH (Version 1.2.7)^70^. The merged raw tags were filtered and developed into clean tags according to QIIME (Version 1.7.0) quality controlled process^71,72^. Then, clean tags were compared with the reference Gold database (Release 20,110,519) and chimera sequence was detected and deleted by using UCHIME Algorithm (Version 7.0.1001)^73,74^. The non-chimera clean effective tags were clustered into OTUs with ≥ 97% similarity by Uparse software (Version 7.0.1001)^75^. The representative sequence for each OTU was selected and the taxonomic information was annotated using SILVA database^76,77^. The procedure of sequencing and annotation was performed at Novogene Bioinformatics Technology Co., Ltd.

Sequencing of the PCR products obtained by amplification of the V3-V4 region of the 16S rRNA gene from the 74 faecal samples analyzed yielded an average of ~ 70,000 filtered partial sequences per sample and a final number of 1691total OTUs.

### Quantification of gut microbiota phyla by qPCR

Absolute levels of the three main gut microbial phyla in our study population (Actinomycetota, Bacillota and Bacteroidota) were determined by qPCR using the primers indicated in Supplementary Table S5 online. PCR reactions were performed in sealed 96 well plates (Applied Biosystems, Foster City, CA) in a 7500 Fast Real Time PCR System (Applied Biosystems). Each reaction was run in a final volume of 25 µL with 1 µL of fecal DNA as template, 0.2 µM of each primer and 2X SYBRGreen PCR Master MIX (Applied Biosystems). Thermal cycling conditions were 95 °C 10 min, followed by 40 cycles of 95 °C 15 s and 1 min at the appropriate primer-pair temperature (see Supplementary Table S5 online). A standard curve for each phylum was generated from serial dilutions of a known quantity of genomic DNA from the most representative bacterium for each phylum. Data were presented as a logarithm with base 10 of colony-forming units (CFUs) per gram of feces.

### Fecal short chain fatty acids (SCFA) measurement

SCFA, including acetic, propionic, butyric, isovaleric, isobutyric, valeric and caproic acids were detected and quantified by Gas Chromatography (GC) as described elsewhere^78^. Briefly, cell free supernatants obtained from the homogenized feces (0.250 mL) were mixed with 0.3 mL methanol, 0.05 mL of 20% formic acid (v/v) and 0.05 mL of an internal standard solution (2-ethylbutyric 1.05 mg/mL). This mixture was centrifuged, and the supernatant was used for quantification of SCFA by GC using a system composed of a 6890NGC injection module (Agilent Technologies Inc., Palo Alto, Ca, USA) connected to a flame injection detector (FID) (Agilent). SCFA concentrations are given as mM. Molar proportions were calculated in some cases by referring the concentration of each SCFA to the total of concentration of all SCFA, considered this as 100%.

### Statistical analysis

Statistical analyses of the results were performed using IBM SPSS version 26.0 (IBM SPSS, Inc., Chicago, IL, United States). Data for all the variables analysed were compared among the three groups of children (ASD, SIB, and GP) considered as such all together (ASD, SIB, GP) or stratified by age (4–7 years, 8–10 years), as well as between ASD subgroups according to the severity of the disease (autistic with mild to moderate ASD, autistic with severe ASD). Shapiro–Wilk test was used to verify the normal distribution of the data. Overall, we used a Kruskal–Wallis test (not normally distributed data) or an ANOVA test (normally distributed data) to test for differences between the three groups of children (ASD, SIB, GP) as such, and stratified by age (4–7 years, 8–10 years). A Mann–Whitney U test (not normally distributed data) or a two-tailed Student’s t-test (normally distributed data) was used to test differences between autistic children’s subgroups (autistic with mild to moderate ASD, autistic with severe ASD). More precisely, to assess differences in the bacterial community composition not normally distributed among groups (ASD, SIB and GP), a Kruskal–Wallis test followed by a post hoc Dunn’s test for pairwise comparison was performed. Mann–Whitney U test was used to compare the microbiota of autistic children according to the severity of the disease. Alpha diversity indices, qPCR results and SCFA data were compared among the three groups of children by using Kruskal–Wallis test followed by a post hoc Dunn’s test for the non-normally distributed data or by using an ANOVA test followed by a post hoc Tukey test for the normally distributed data. A two-tailed Student’s t-test (normally distributed data) or Mann–Whitney U test (non-normally distributed data) was used to compare alpha diversity indices and SCFA levels between autistic children’s subgroups (autistic with mild to moderate ASD, autistic with severe ASD). To compare microbial communities (beta diversity), a square matrix of distance was calculated and visualized with Non-Metric Multi-Dimensional Scaling (NMDS) by QIIME software (Version 1.7.0) and Adonis test was performed by R software (Vegan package: adonis function) to conduct comparisons among groups. LEfSe (linear discriminant analysis effect size) analysis was conducted to estimate the taxa discriminating significantly among the three groups of study by using a Kruskal–Wallis sum-rank test (alpha significance level of 0.05) and a Wilcoxon test (alpha significance level of 0.05) for pairwise comparison, followed by an LDA (Logarithmic Linear Discriminant Analysis) to estimate the effect-size (threshold of 2) by LEfSe software^25^. Alpha diversity was studied by Chao 1 and Shannon indices calculated with QIIME (Version 1.7.0). All statistical analyses were two sided and a *p*-value < 0.05 was considered as statistically significant. Unless otherwise specified, data were expressed as mean ± standard deviation. Relative abundances were used with a mean prevalence threshold of ≥ 0.05% of abundance in at least one group of children.

### Supplementary Information

Supplementary Information.

## Data Availability

The data analyzed in this study are included in this published article (and its Supplementary Information files). The sequencing datasets used in this study are publicly available in the NCBI Sequence Read Archive (SRA), BioProject ID: PRJNA972983.
