# Supplementary material for: Age and severity-dependent gut microbiota alterations in Tunisian children with autism spectrum disorder
Source: Sci Rep. 2023 Oct 25;13:18218. doi: 10.1038/s41598-023-45534-0 (PMC10600251; doi:10.1038/s41598-023-45534-0)
Supplement: Supplementary file 1 — Supplementary Information. [file 41598_2023_45534_MOESM1_ESM.docx]

**Supplemental information**

Age and severity-dependent gut microbiota alterations in Tunisian children with autism spectrum disorder

Mariem Chamtouri^1,2^, Naoufel Gaddour^3^, Abderrahmen Merghni^4^, Maha Mastouri^2^, Silvia Arboleya^1,5, *^, Clara G. de los Reyes-Gavilán^1,5, *^

^1^ Department of Microbiology and Biochemistry of Dairy Products, Instituto de Productos Lácteos de Asturias (IPLA-CSIC), 33300 Villaviciosa, Spain

^2^ Laboratory of Transmissible Diseases and Biologically Active Substances LR99ES27, Faculty of Pharmacy, University of Monastir, Monastir 5000, Tunisia

^3^ Unit of Child Psychiatry, Monastir University Hospital, Monastir 5000, Tunisia

^4^ Laboratory of Antimicrobial Resistance LR99ES09, Faculty of Medicine of Tunis, University of Tunis El Manar, Tunis 1068, Tunisia

^5^ Diet, Microbiota, and Health Group, Instituto de Investigación Sanitaria del Principado de Asturias (ISPA), 33011 Oviedo, Spain

^*^Correspondence: silvia.arboleya@ipla.csic.es; greyes_gavilan@ipla.csic.es

Figure S1. Absolute levels (log_10_ CFU/g feces) of fecal microbial phyla (Actinomycetota, Bacillota, Bacteroidota) determined by qPCR in samples from autistic children (ASD), siblings (SIB) and children from the general population (GP). (*) indicates statistically significant differences (p<0.05) between GP and SIB groups. Bars represent mean values and lines the standard deviation.

Figure S2. Comparison of Bifidobacteriaceae/Coriobacteriaceae ratio (relative abundances) between autistic children (ASD), their siblings (SIB) and children from the general population (GP) as such, stratified by age (4-7 years, 8-10 years) and between autistic children's subgroups (children with mild to moderate ASD, children with severe ASD). Bars represent mean values and lines the standard deviation. Statistically significant differences (p<0.05) are indicated by (*).

Table S1. Relative abundance of statistically significant bacterial genera identified by sequencing the V3-V4 region of the 16S rRNA gene in fecal samples from autistic children (ASD), their siblings (SIB) and children from the general population (GP). Values indicate means ± standard deviation and letters (a, b) at the right of numerical values indicate significant differences (p <0.05) among groups.

|  | **ASD** | **SIB** | **GP** | **p-value** |
| --- | --- | --- | --- | --- |
| *Collinsella* | 18.16±6.03^a^ | 15.09±9.03^ab^ | 12.54±7.74^b^ | 0.011 |
| *Subdoligranulum* | 3.96±2.49^a^ | 5.60±2.98^b^ | 3.18±1.84^a^ | 0.005 |
| *Eggerthella* | 0.09±0.09^a^ | 0.16±0.15^b^ | 0.66±2.54^b^ | 0.022 |
| *Intestinibacter* | 2.16±1.21^ab^ | 3.72±3.39^a^ | 1.76±1.11^b^ | 0.038 |
| *Coriobacteriaceae_UCG-003_group* | 1.06±2.38^a^ | 0.51±1.72^ab^ | 0.41±1.56^b^ | 0.018 |
| *Ruminococcus_torques_ group* | 1.12±1.44^a^ | 0.86±0.44^a^ | 0.39±0.16^b^ | 0 |
| *Senegalimassilia* | 2.07±1.90^a^ | 1.05±0.98^b^ | 1.23±1.12^ab^ | 0.05 |
| *Ruminococcaceae_CAG-352_group* | 0.15±0.28^a^ | 0.34±0.43^b^ | 0.58±1.26^b^ | 0.002 |
| *Klebsiella* | 0.04±0.05^a^ | 0.31±0.95^ab^ | 0.08±0.12^b^ | 0.024 |
| *Desulfovibrio* | 0.02±0.04^a^ | 0.01±0.01^a^ | 0.32±0.76^b^ | 0.001 |
| *Eubacterium_halli_group* | 0.91±0.36^a^ | 1.25±0.63^b^ | 0.79±0.29^a^ | 0.011 |
| *Haemophilus* | 0.02±0.11^a^ | 0.00±0.01^a^ | 0.12±0.52^b^ | 0.008 |
| *Fusicatenibacter* | 0.51±0.26^a^ | 1.09±0.72^b^ | 0.58±0.31^a^ | 0.005 |
| *Sarcina* | 0.51±0.66^a^ | 0.15±0.15^b^ | 0.16±0.28^b^ | 0.004 |
| *Eubacterium siraeum_group* | 0.02±0.03^a^ | 0.06±0.16^ab^ | 0.15±0.47^b^ | 0.05 |
| *Roseburia* | 0.14±0.11^a^ | 0.33±0.47^b^ | 0.15±0.15^a^ | 0.031 |
| *Butyricicoccus* | 0.17±0.15^a^ | 0.31±0.42^ab^ | 0.33±0.27^b^ | 0.029 |
| *Slackia* | 0.42±0.34^a^ | 0.54±0.51^a^ | 0.28±0.41^b^ | 0.018 |
| *Erysipelatoclostridium* | 0.02±0.03^a^ | 0.03±0.05^ab^ | 0.11±0.30^b^ | 0.028 |
| *Monoglobus* | 0.16±0.09^a^ | 0.33±0.21^b^ | 0.33±0.31^b^ | 0.002 |
| *Oscillospiraceae_NK4A214_group* | 0.15±0.13^a^ | 0.21±0.19^ab^ | 0.29±0.26^b^ | 0.03 |
| *Gordonibacter* | 0.08±0.17^a^ | 0.17±0.22^b^ | 0.17±0.27^b^ | 0.03 |
| *Bacteroides* | 0.10±0.25^a^ | 0.07±0.11^a^ | 0.17±0.21^b^ | 0.003 |
| *Lachnoclostridium* | 0.05±0.040^a^ | 0.070±0.09^a^ | 0.15±0.20^b^ | 0.001 |
| *Gluconobacter* | 0^a^ | 0^a^ | 0.19±0.33^b^ | 0 |
| *Pseudomonas* | 0^a^ | 0^a^ | 0.36±0.28^b^ | 0 |
| *Citrobacter* | 0.01±0.01^a^ | 0.06±0.21^a^ | 0.04±0.04^b^ | 0 |
| *Solobacterium* | 0.09±0.21^a^ | 0.02±0.02^b^ | 0.03±0.09^b^ | 0.039 |
| *Eubacterium_eligens_group* | 0.04±0.040^a^ | 0.06±0.06^b^ | 0.09±0.14^b^ | 0.008 |
| *Ruminococcus_gnavus_group* | 0.01±0.02^a^ | 0.01±0.22^a^ | 0.08±0.14^b^ | 0 |
| *Kroppenstedtia* | 0^a^ | 0^a^ | 0.06±0.10^b^ | 0 |
| *Acinetobacter* | 0^a^ | 0^a^ | 0.06±0.09^b^ | 0 |
| *Actinomyces* | 0.06±0.03^a^ | 0.08±0.04^b^ | 0.09±0.06^b^ | 0.015 |
| *Lachnospircaeae_FCS020_group* | 0.08±0.71^ab^ | 0.10±0.06^a^ | 0.05±0.03^b^ | 0.006 |
| *Megamonas* | 0^a^ | 0^a^ | 0.05±0.07^b^ | 0 |
| *Methanosphera* | 0.08±0.23^a^ | 0.00±0.01^b^ | 0.01±0.45^b^ | 0.006 |
| *Colidextribacter* | 0.03±0.05^a^ | 0.02±0.03^ab^ | 0.05±0.07^b^ | 0.04 |
| *Lachnospiraceae_NK3A20_group* | 0.05±0.18^a^ | 0.00±0.01^ab^ | 0^b^ | 0 |

Table S2. Relative abundance of statistically significant bacterial genera identified by sequencing the V3-V4 region of the 16S rRNA gene in fecal samples from autistic children (ASD), their siblings (SIB) and children from the general population (GP) aged between 4-7 years. Values indicate means ± standard deviation and letters (a, b) at the right of numerical values indicate significant differences (p <0.05) among groups.

|  | **ASD 4-7** | **SIB 4-7** | **GP 4-7** | **p-value** |
| --- | --- | --- | --- | --- |
| *Bifidobacterium* | 28.70±16.72^a^ | 27.07±7.86^a^ | 39.46±13.95^b^ | 0.036 |
| *Collinsella* | 20.84±4.92^a^ | 13.83±7.72^b^ | 12.52±5.59^b^ | 0.016 |
| *Subdoligranulum* | 3.66±2.21^a^ | 5.57±1.81^b^ | 2.72±1.14^a^ | 0.001 |
| *Coriobacteriaceae_UCG-003* | 0.12±0.08^a^ | 0.78±2.19^a^ | 0.06±0.09^b^ | 0.043 |
| *Ruminococcus_torques_ group* | 1.86±2.28^a^ | 0.88±0.38^a^ | 0.35±0.14^b^ | 0 |
| *Christensenellaceae_R-7_ group* | 0.52±0.35^a^ | 1.25±0.85^b^ | 1.58±2.14^ab^ | 0.046 |
| *Desulfovibrio* | 0.00±0.00^a^ | 0.01±0.01^ab^ | 0.47±1.02^b^ | 0.012 |
| *Turicibacter* | 0.57±0.95^ab^ | 0.41±0.30^a^ | 0.18±0.10^b^ | 0.032 |
| *Haemophilus* | 0.00±0.00^a^ | 0.01±0.01^a^ | 0.23±0.73^b^ | 0.022 |
| *Sarcina* | 0.49±0.61^a^ | 0.16±0.15^ab^ | 0.07±0.07^b^ | 0.019 |
| *Eubacterium_siraeum_group* | 0.01±0.02^a^ | 0.09±0.20^b^ | 0.21±0.66^b^ | 0.034 |
| *Oscillospiraceae_NK4A214_group* | 0.11±0.14^a^ | 0.24±0.22^ab^ | 0.27±0.16^b^ | 0.047 |
| *Lachnoclostridium* | 0.05±0.05^a^ | 0.08±0.11^a^ | 0.13±0.11^b^ | 0.02 |
| *Gluconobacter* | 0^a^ | 0^a^ | 0.13±0.25^b^ | 0.005 |
| *Pseudomonas* | 0^a^ | 0^a^ | 0.36±0.27^b^ | 0 |
| *Citrobacter* | 0.01±0.01^a^ | 0.09±0.26^ab^ | 0.05±0.05^b^ | 0.006 |
| *Eubacterium_eligens_group* | 0.03±0.05^a^ | 0.06±0.04^b^ | 0.07±0.07^b^ | 0.02 |
| *Ruminococcus_gnavus_group* | 0.01±0.01^a^ | 0.01±0.03^a^ | 0.07±0.10^b^ | 0.005 |
| *Kroppenstedtia* | 0^a^ | 0^a^ | 0.06±0.10^b^ | 0 |
| *Acinetobacter* | 0^a^ | 0^a^ | 0.05±0.07^b^ | 0 |
| *Lachnospircaeae_FCS020_group* | 0.11±0.11^ab^ | 0.10±0.05^a^ | 0.05±0.03^b^ | 0.013 |
| *Megamonas* | 0^a^ | 0^a^ | 0.07±0.09^b^ | 0 |
| *Colidextribacter* | 0.01±0.01^a^ | 0.02±0.01^ab^ | 0.04±0.04^b^ | 0.025 |
| *Lachnospiraceae_NK3A20_group* | 0.02±0.01^a^ | 0^b^ | 0^b^ | 0 |

Table S3. Relative abundance of statistically significant bacterial genera identified by sequencing the V3-V4 region of the 16S rRNA gene in fecal samples from autistic children (ASD), their siblings (SIB) and children from the general population (GP) aged between 8-10 years. Values indicate means ± standard deviation and letters (a,b) at the right of numerical values indicate significant differences (p <0.05) among groups.

|  | **ASD 8-10** | **SIB 8-10** | **GP 8-10** | **p-value** |
| --- | --- | --- | --- | --- |
| *Dialister* | 1.41±2.30^a^ | 0.26±0.29^b^ | 1.20±1.99^ab^ | 0.009 |
| *Ruminococcus_torques_ group* | 0.71±0.25^a^ | 0.82±0.57^a^ | 0.44±0.16^b^ | 0.014 |
| *Ruminococcaceae_CAG-352_group* | 0.11±0.17^a^ | 0.15±0.11^ab^ | 0.88±1.75^b^ | 0.02 |
| *Prevotella* | 0.38±0.93^a^ | 0.12±0.15^a^ | 0.66±0.76^b^ | 0.017 |
| *Desulfovibrio* | 0.02±0.05^a^ | 0.01±0.00^a^ | 0.16±0.32^b^ | 0.013 |
| *Eubacterium_siraeum_group* | 0.03±0.03^ab^ | 0.01±0.02^a^ | 0.09±0.1^b^ | 0.034 |
| *Gastranaerophilales* | 0.17±0.49^a^ | 0.02±0.03^ab^ | 0.01±0.01^b^ | 0.013 |
| *Monoglobus* | 0.14±0.07^a^ | 0.34±0.25^b^ | 0.28±0.22^b^ | 0.016 |
| *Bacteroides* | 0.10±0.29^a^ | 0.02±0.02^a^ | 0.19±0.28^b^ | 0.006 |
| *Lachnoclostridium* | 0.05±0.04^a^ | 0.06±0.05^ab^ | 0.18±0.26^b^ | 0.04 |
| *Gluconobacter* | 0^a^ | 0^a^ | 0.26±0.38^b^ | 0.002 |
| *Pseudomonas* | 0^a^ | 0.00±0.00^a^ | 0.36±0.32^b^ | 0 |
| *Citrobacter* | 0.01±0.01^a^ | 0.01±0.01^a^ | 0.04±0.03^b^ | 0 |
| *Solobacterium* | 0.13±0.26^a^ | 0.01±0.02^b^ | 0.05±0.12^ab^ | 0.024 |
| *Ruminococcus_gnavus_group* | 0.02±0.03^a^ | 0.01±0.00^a^ | 0.09±0.17^b^ | 0.007 |
| *Eggerthellaceae_ CHKCI002_group* | 0.01±0.02^a^ | 0.06±0.15^ab^ | 0.09±0.12^b^ | 0.023 |
| *Ruminococcus* | 0.11±0.10^a^ | 0.08±0.03^a^ | 0.14±0.08^b^ | 0.05 |
| *Kroppenstedtia* | 0^a^ | 0^a^ | 0.06±0.1^b^ | 0 |
| *Acinetobacter* | 0^a^ | 0.00±0.00^a^ | 0.07±0.1^b^ | 0 |
| *Actinomyces* | 0.05±0.02^a^ | 0.05±0.02^a^ | 0.10±0.08^b^ | 0.009 |
| *Megamonas* | 0^a^ | 0.00±0.00^a^ | 0.04±0.05^b^ | 0 |
| *Methanosphera* | 0.12±0.29^a^ | 0.00±0.01^b^ | 0.01±0.05^b^ | 0.025 |
| *Lachnospiraceae_NK3A20_group* | 0.06±0.23^a^ | 0.01±0.02^a^ | 0^b^ | 0.043 |

Table S4**.** Relative abundance of statistically significant bacterial genera identified by sequencing the V3-V4 region of the 16S rRNA gene in fecal samples from autistic children's subgroups: children with mild to moderate ASD and children with severe ASD. Values indicate means ± standard deviation.

|  | **Mild to moderate ASD** | **Severe ASD** | **p-value** |
| --- | --- | --- | --- |
| *Bifidobacterium* | 39.16±12.39 | 26.66±14.54 | 0.01 |
| *Libanicoccus* | 0.70±1.15 | 3.23 | 0.041 |
| *Catenibacterium* | 1.52±3.40 | 2.67±2.24 | 0.046 |
| *Clostridia_UCG-014* | 0.50±0.68 | 0.97±0.82 | 0.016 |
| *Senegalimassilia* | 1.19±0.95 | 2.64±2.16 | 0.041 |
| *Enterorhabdus* | 0.20±0.21 | 0.86±0.85 | 0.027 |
| *Monoglobus* | 0.11±0.06 | 0.18±0.09 | 0.046 |
| *Oscillospiraceae_UCG-005* | 0.09±0.07 | 0.17±0.13 | 0.04 |

Table S5. Primers and annealing temperatures used for qPCR in this study

| Target | Primer sequence (5'-3') | Annealing Tª (°C) | Reference |
| --- | --- | --- | --- |
| Actinomycetota | Act920F3: TACGGCCGCAAGGCTA  Act1200R: TCRTCCCCACCTTCCTCCG | 61.5 | [Bacchetti De Gregoris et al. 2011] |
| Bacillota | Firm934F: GGAGYATGTGGTTTAATTCGAAGCA  Firm1060R: AGCTGACGACAACCATGCAC | 60 | [Guo et al. 2008] |
| Bacteroidota | Bact934F: GGARCATGTGGTTTAATTCGATGAT  Bact1060R: AGCTGACGACAACCATGCAG | 60 | [Guo et al. 2008] |

**References:**

Bacchetti De Gregoris, T., Aldred, N., Clare, A. S.& Burgess, J. G. Improvement of phylum- and class-specific primers for real-time PCR quantification of bacterial taxa. *J. Microbiol. Methods*. **86,** 351–6 (2011).

Guo, X. *et al*. Development of a real-time PCR method for Firmicutes and Bacteroidetes in faeces and its application to quantify intestinal population of obese and lean pigs. *Lett. Appl. Microbiol*. **47**, 367-73 (2008).
